# Treatment of acute thoracolumbar burst fractures with kyphoplasty and short pedicle screw fixation: Transpedicular intracorporeal grafting with calcium phosphate: A prospective study

**DOI:** 10.4103/0019-5413.37000

**Published:** 2007

**Authors:** Panagiotis Korovessis, Thomas Repantis, Petsinis George

**Affiliations:** Chief Orthopaedic Department, General Hospital “Agios Andreas”, 1 Tsertidou str., 26224 Patras, Greece

**Keywords:** Balloon kyphoplasty, calcium phosphate, neurological deficit, pedicle screw, short internal fixation, thoracolumbar vertebral fracture, transpedicular grafting

## Abstract

**Background:**

In the surgical treatment of thoracolumbar fractures, the major problem after posterior correction and transpedicular instrumentation is failure to support the anterior spinal column, leading to loss of correction and instrumentation failure with associated complaints. We conducted this prospective study to evaluate the outcome of the treatment of acute thoracolumbar burst fractures by transpedicular balloon kyphoplasty, grafting with calcium phosphate cement and short pedicle screw fixation plus fusion.

**Materials and Methods:**

Twenty-three consecutive patients of thoracolumbar (T_9_ to L_4_) burst fracture with or without neurologic deficit with an average age of 43 years, were included in this prospective study. Twenty-one from the 23 patients had single burst fracture while the remaining two patients had a burst fracture and additionally an adjacent A1-type fracture. On admission six (26%) out of 23 patients had neurological deficit (five incomplete, one complete). Bilateral transpedicular balloon kyphoplasty with liquid calcium phosphate to reduce segmental kyphosis and restore vertebral body height and short (three vertebrae) pedicle screw instrumentation with posterolateral fusion was performed. Gardner kyphosis angle, anterior and posterior vertebral body height ratio and spinal canal encroachment were calculated pre- to postoperatively.

**Results:**

All 23 patients were operated within two days after admission and were followed for at least 12 months after index surgery. Operating time and blood loss averaged 45 min and 60 cc respectively. The five patients with incomplete neurological lesions improved by at least one ASIA grade, while no neurological deterioration was observed in any case. The VAS and SF-36 (Role physical and Bodily pain domains) were significantly improved postoperatively. Overall sagittal alignment was improved from an average preoperative 16° to one degree kyphosis at final followup observation. The anterior vertebral body height ratio improved from 0.6 preoperatively to 0.9 (*P*<0.001) postoperatively, while posterior vertebral body height improved from 0.95 to 1 (*P*<0.01). Spinal canal encroachment was reduced from an average 32% preoperatively to 20% postoperatively. Cement leakage was observed in four cases (three anterior to vertebral body and one into the disc without sequalae). In the last CT evaluation, there was a continuity between calcium phosphate and cancellous vertebral body bone. Posterolateral radiological fusion was achieved within six months after index operation. There was no instrumentation failure or measurable loss of sagittal curve and vertebral height correction in any group of patients.

**Conclusions:**

Balloon kyphoplasty with calcium phosphate cement secured with posterior short fixation in the thoracolumbar spine provided excellent immediate reduction of posttraumatic segmental kyphosis and significant spinal canal clearance and restored vertebral body height in the fracture level.

Traditional pedicle screw instrumentation allows indirect reduction and kyphosis correction of thoracolumbar burst^1^,^2^ by distraction and ligamentotaxis, but because of frequent failure to support the anterior spinal column, loss of correction associated with high rate of failure is not rare.^3^–^7^ Consequently, anterior instrumentation with strut grafting, mesh cage and plates have been introduced to augment the anterior vertebral column and have proved to be effective. However, an anterior approach is more invasive and is associated with prolonged operation and hospitalization time, blood loss, donor site complaints and increased morbidity.^8^,^9^

Transpedicular cancellous bone grafting to the fractured body has been attempted as a method to provide significant additional support to the anterior column but it was abandoned because of a significant failure rate.^10^–^15^ Recently, transpedicular intracorporeal hydroxyapatite stick grafting associated with short pedicle screw fixation has been successfully used to treat burst fractures.^16^ An increasing number of young patients with acute traumatic compression and burst fractures have recently undergone kyphoplasty. Kyphoplasty with polymethylmethacrylate (PMMA) has serious drawbacks^17^–^21^ and thus it does not appear to be a suitable cement for this young patient population. The ideal bone cement would rather be a compatible, osteoconductive, slowly biodegradable (resorbable) augmentation material amenable to replacement by newly formed bone.

The hypothesis of this prospective clinical investigation in patients with fresh thoracolumbar burst fractures was that transpedicular vertebral body fracture reduction with balloon kyphoplasty with calcium phosphate cement supplemented with short pedicle screw fixation, could sufficiently and permanently reduce lost fractured body height, segmental kyphosis and decrease spinal canal encroachment in biologic young patients.

## MATERIALS AND METHODS

From May 2004 through April 2005, 29 individuals, who were diagnosed to have one thoracolumbar burst fracture (T_9_-L_4_) with or without neurological deficit were identified and evaluated for participation in a prospective study. The internal review board approval was undertaken. Eligible patients were required to sign an informed consent form. The inclusion criteria for this study were burst thoracolumbar fracture (AO-type A_3,3_), load Sharing Classification Grade >6 and early operation (within two days following trauma). The exclusion criteria were: polytraumatized patients with severe osteoporosis (plain roentgenograms), patients with preexisting spinal deformity (scoliosis, previous vertebral fracture in the area of interest), degenerative or other spinal stenosis and patients with previous spinal operation. Twenty-three out of the 29 patients who met the inclusion criteria were enrolled in this study. This series included three women and 20 men age ranging from 25 to 63 years, (with an average age ± SD 43±7 years) at surgery. The mechanism of injury was fall from a height in 17 patients and road traffic accident in the remaining six patients. Non-serious associated injuries (colles, calcaneus and nondisplaced pubic rami fractures, rib fractures) were recorded in six patients but they did not require additional surgery. Neurological assessment was made for each patient using a rating system based on that of the American Spine Injury Association (ASIA) impairment scale. Back pain intensity was recorded on VAS (10-point scale). Functional outcome was measured using the SF-36 domains Role Physical and Bodily pain.

Supine anteroposterior and lateral supine roentgenograms of the spine were taken on admission in all patients [Figure 1a]. The computed tomography (CT) was performed in all patients and MRI (when available) before and after surgery [Figure 1c–d]. All patients were followed radiographically after surgery by standing anteroposterior and lateral roentgenograms [Figure 1b] of the whole spine and supine oblique roentgenograms and sitting lateral flexion-extension roentgenograms to assess fusion and stability of the fused spine. Computed tomography scans, including reconstruction images, were taken immediately after surgery, at six months and then once a year. The variables that were evaluated on plain radiographs were: Gardner segmental kyphotic deformity^22^ (formed from the line of the upper endplate of the non-fractured vertebra above the fractured vertebra the line of the lower endplate of the fractured vertebra), anterior vertebral body height ratio (AVBHr)^22^ (the average of the anterior vertebral body of the vertebrae above and below the fractured vertebra divided by the anterior vertebral body height of the fractured vertebra) and posterior vertebral body height ratio (PVBHr)^22^ (the average of the posterior vertebral body of the vertebrae above and below the fractured vertebra divided by the posterior vertebral body height of the fractured vertebra). Spinal canal encroachment (the AP diameter of the spinal canal at the level of maximum retropulsion divided by the average AP spinal canal diameter of the intact vertebrae above and below the fractured vertebrae) and clearance (the AP diameter of the spinal canal free of retropulsed bone divided by the average AP spinal canal diameter of the intact vertebrae above and below the fractured vertebrae) were calculated pre- and one year postoperatively on CT scans.^22^ Patients were encouraged to walk while wearing a three-point fixation brace immediately following day after surgery for one month. Strenuous physical activity was restricted up to 12 weeks after surgery. Statistical analysis was performed with paired t-test for changes of each radiographic parameter and with correlation coefficient (R) for comparison between changes of different parameters.

**Figure 1a:**
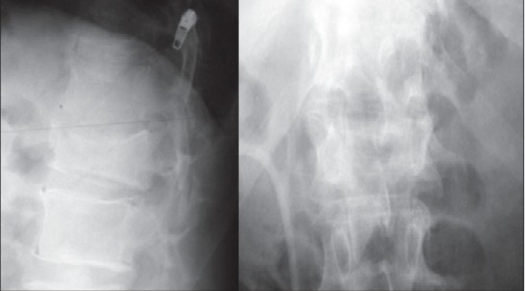
X-ray lumbar spine (AP and Lateral) in a 48-year-old male patient showing a burst fracture of L_2_

**Figure 1b:**
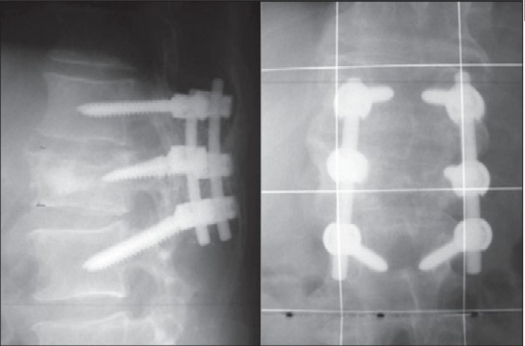
X-ray lumbar spine (Lateral and AP) of the same patient three months postoperatively showing excellent alignment following short fixation

**Figure 1c,d:**
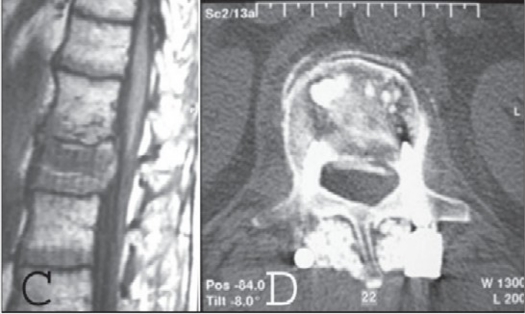
T1WI of preoperative MRI of same patient shows spinal canal narrowing at the level L_2_ (c) and postoperative CT scan (d) of the same patient at the level L_2_ showing calcibon on the anterior half of the vertebral body

### Surgical technique

The patients were operated under general anesthesia in a prone position on the Acromed frame on a radiolucent Maquet operating table. Somatosensory and Motor Evoked Potentials and Triggered EMG were recorded intraoperatively in all patients except those with ASIA A. The spontaneous reposition of the segmental kyphosis and subsequently spinal canal encroachment from the retropulsed fragments on the Acromed frame was then controlled by image intensifier. The instrumentation construct was short i.e. three vertebrae, including intermediate screw for the fractured vertebra. The standard posterior midline approach was used and pedicle screws SYNERGY (Biomet Spine, NJ, USA) of appropriate diameter were placed into the pedicles of adjacent vertebrae above and below the fractured vertebra. Posterior wall decompression was not achieved via ligamentotaxis but merely by positioning of the patients and appropriate longitudinal rod contouring^23^ without applying distraction along the entire instrumentation. Following screw insertion and under biplane image intensifier control and continuous EMG monitoring, kyphoplasty was performed. A 2-mm diameter trokar (Kyphon, Inc, Sunnyvale, CA, USA) was inserted into both pedicles of the fractured vertebra according to manufacturer's instructions. The position of the cannula was continuously controlled in both planes and then enlarged with the use of an access cannula with a trokar and gentle reaming. Once the cannula reached the middle third of the fractured vertebral body, the void was reamed and two kyphoplasty balloons were carefully inserted and inflated under continuous fluoroscopic monitoring. In the first five cases, before balloon inflation we performed intraoperative myelography with a 22-gauge fine needle to observe any posterior displacement of bone fragments into the spinal canal. Subsequently, Calcibon, (Biomet, Wehrheim, Germany), which is a calcium phosphate cement in a radiopaque liquid that hardens quickly, was injected anteriorly into the void produced by the balloon inflation. Subsequently, two 30-mm pedicle screws were inserted in the pedicle of the fractured vertebra through which kyphoplasty was performed to increase the stability of the construct and to decrease local kyphosis. No open reduction, decompression or laminectomies or laminotomies or manipulation of the vertebral body fracture fragments producing vertebral canal encroachment were performed. The posterolateral fusion using local bone chips derived from meticulous decortication of the posterior vertebral elements within the instrumented area, mixed with calcium phosphate granules (Calcibon, Biomet, Wehrheim Germany) were used. Calcibon is a synthetic material that consists of two parts: a powder and a liquid part. Powder consists of Tricalcium Phosphate, Calciumhydrogenphosphate, Calciumcarbonat and Hydroxyapatite and the liquid part of dinatrium hydrogenphosphate. If liquid and powder are mixed in an appropriate ratio they form a paste, which, at room or body temperature, sets by precipitation of one or more other solid compounds, of which at least one is a calcium phosphate.^24^

## RESULTS

The mean operative time was 45 min (range, 35-50 min). The total blood loss (intraoperatively plus after surgery to maintain Hb >8 g) was 600 ml (range, 200-750 ml). The maximum intravertebral pressure of the balloons varied between 150 and 200 psi. These values were within the safe operating range for the balloons (Kyphon, inc, Sunnyvale, CA, USA). The volume of injected Calcibon required for fluoroscopically complete filling of the defect per vertebra ranged from 3 to 6 g. All patients were observed clinically and radiologically for a minimum of 12 months, with an average followup of 21 months (range 12-31 months) [Figure 1e]. Based on the ASIA neurological grading system, one patient had Grade A, two patients had Grade C, three had Grade D and 17 had Grade E on admission. All five patients with incomplete neurological impairment had at least one ASIA grade neurological improvement on final followup observation.

**Figure 1e:**
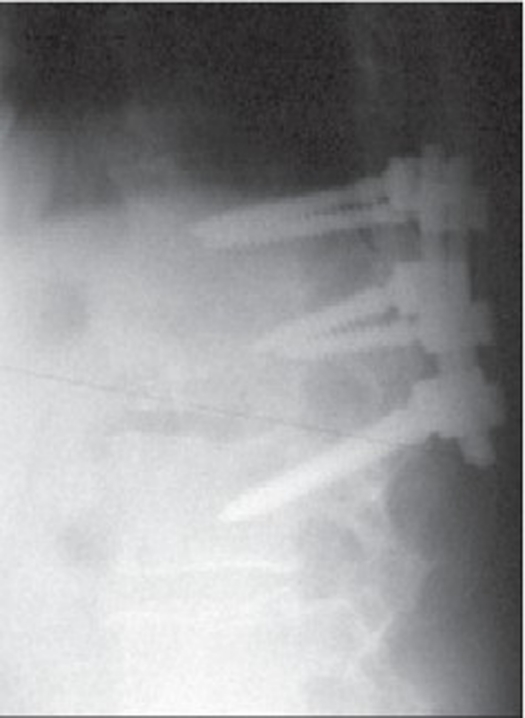
Lateral roentgenogram of the same patient 31 months postoperatively showing Calcibon incorporation

The domain role physical improved from an average±SD 35 ± 15 preoperatively to 80 ± 20 postoperatively (*P*<0.001) and the domain bodily pain from an average±SD 25±11 preoperatively to 85 ±15 postoperatively (*P*<0.001), at the six months followup. No further changes in the SF-36 scores in both domains were reported. The preoperative (back pain) VAS score averaged, SD 9±1 and improved postoperatively at the latest evaluation to a score of 3.4 ± 2.3.

The hospital stay for the patients averaged two days (range, one to four days). Preoperative Gardner angle was reduced from an average +SD 16° + 11°, to 1° + 5° (correction 94%) postoperatively.

Preoperative Spinal canal encroachment was reduced from an average +SD 32% + 21%, to 20% + 18% (correction 37.5%) postoperatively. Preoperative AVBHr was reduced from an average +SD 0.6 ± 0.13 to 0.92 ± 0.08 (correction 53%) postoperatively. Preoperative PVBHr was reduced from an average +SD 0.95 ± 0.06 to 0.98 ± 0.03 (3% correction postoperatively Preoperative spinal canal encroachment was significantly correlated with Gardner angle (R=0.53, *P*<0.05) and AVBHr (R=-0.63, *P*<0.02). Preoperative Gardner angle was significantly correlated with AVBHr (R=-0.739, *P*<0.01).

Final radiographs and CT scan showed radiologically healed fractures within six months postoperatively with posterolateral intertransverse fusion. Cement leakage occurred in four cases (three anterior to the vertebral body and one into the disc), however, without any sequalae of clinical importance. No patient had postoperative neural deterioration. Superficial wound necrosis developed in one patient and healed without additional surgery. We did not encounter any implant failure, infection and no patient needed additional surgery.

## DISCUSSION

Burst fracture is a two- or three-column injury^25^ that may lead to a 70% decrease of flexural stiffness and 70% decrease of torsional stiffness.^26^,^27^ Thus, restoration of both anterior and middle columns seems to be mandatory to safeguard stability of the injured spine. Posterior, anterior and combined operative techniques have been used for operative stabilization of thoracolumbar burst fractures. Anterior decompression allows decompression under direct vision,^9^,^28^–^30^ however, anterior approach is associated with increased morbidity as compared to posterior approach.^23^,^31^,^32^ Recent studies showed no clear advantage when anterior was compared with posterior surgery.^33^,^34^ Most surgeons prefer posterior instrumentation devices since most of them are familiar with these techniques.

Conventional pedicle-screw instrumentations use distraction forces to indirectly reduce kyphotic sagittal deformity and restore vertebral body height by “ligamentotaxis” with failure rates of 9-54%.^3^,^35^–^44^ Following posterior distraction, canal decompression is limited and often incomplete, while when distraction is applied in the lumbar spine it can result in flattening of lumbar lordosis. This led some surgeons to advocate combined anterior and posterior approaches.^22^,^23^,^32^,^45^–^53^

The transpedicular grafting was introduced to increase the stiffness of the fractured vertebral body,^1^,^2^,^10^,^38^,^53^–^59^ although a correction loss^5^,^41^,^60^ between 2° and 10° was reported or warned for a potentially dangerous situation if graft was not placed carefully.^11^–^14^ Transpedicular augmentation techniques (kyphoplasty and vertebroplasty) are gaining importance for treatment of vertebral compression fractures. The self-hardening calcium phosphate bone cements have been developed as an alternative to overcome possible longterm side-effects of PMMA.^61^–^63^ The majority of patients with traumatic vertebral fractures are aged between 20 and 50 years, hence the use of more biocompatible bone cements (hydroxyapatite, calcium phosphate etc) is advocated.^16^,^64^,^65^

Calcium phosphate cements are biocompatible materials without local heating or toxic effects on surrounding bone tissue and being bioactive they degrade over time by creeping substitution^66^–^68^ and can stimulate formation of new bone substance at the bone-cement interface (osteoconductivity).^69^ The absorption of PMMA versus that of calcium phosphate was compared and showed a partial absorption of calcium phosphate by osteoclasic activity after six months in animals and human beings.^70^–^72^

Hydroxyapatite in solid stick form has been successfully used for transpedicular implantation following^16^ indirect reduction and pedicle screw fixation in acute thoracolumbar burst fractures. The reduction of posttraumatic kyphosis reduced from 20° to -1° lordosis,^16^ while the spinal canal narrowing reduced from 64% to 22%. Although the authors^16^ removed the rods on an average of 12 months after the index operation, the average measured loss of kyphosis correction at the final evaluation was 1° and the spinal canal encroachment was furthermore improved to 11% in the last evaluation. In our series posttraumatic sagittal deformity was reduced to 1° kyphosis together with restoration of AVBHr and PVBHr. Spinal canal encroachment not only did not increase following kyphoplasty, but in contrary it was significantly reduced. This clearly shows the unique advantage of kyphoplasty in safely reducing burst fractures with significant spinal canal encroachment in relatively young patients with non-osteoporotic bone.

In the present study the use of inflatable kyphoplasty balloons created an adequate bone void in the anterior half of the fractured vertebral body, where liquid calcium phosphate was injected under low pressure, thereby decreasing the risk of leakage and undesirable displacement of bone fragments. Verlaan *et al*.,^73^ In a cadaveric model found no clinically relevant displacement of bone anteriorly or posteriorly to spinal canal after balloon kyphoplasty with calcium phosphate. A biomechanical study^74^ on the transpedicular vertebral body reinforcement of thoracolumbar burst fractures with hydroxyapatite cement showed that this augmentation reduced pedicle screw-bending moments and increased initial stiffness in the flexion-extension plane significantly. The preliminary radiological and CT results in the present series justify the results of this mechanical study.^74^ A recent^75^ biomechanical *in vitro* testing of human osteoporotic lumbar vertebrae following prophylactic kyphoplasty with different materials (PMMA, calcium phosphate cement and silicone base material) showed that calcium phosphate cement displayed identical *in vitro* mechanical behavior (similar subsidence behavior in axial compression) to PMMA. The resistance of calcium phosphate to compressive forces has been demonstrated to be not significantly different *in vitro* from PMMA.^67^ This mechanical situation seems to obviate the need for an additional anterior open approach for strut grafting, cages and plating.

Hillmeier *et al*.,^76^ have compared PMMA and Calcibon with balloon kyphoplasty in vertebral body fractures and showed comparable clinical and radiological results and cement leakage. In our series Calcibon leakage was without any sequalae of clinical significance. Furthermore, in the event of calcium phosphate leakage, the damage to surrounding tissue will most likely be less compared with PMMA, by virtue of its isothermic properties during the setting phase.^17^,^77^,^78^ Moreover, the quick hardening process of calcium phosphate and the lack of exothermic reaction seem to be two significant advantages compared to PMMA, particularly in the case of leakage close to vital structures. The grafting with calcium phosphate adds to the augmented fractured body compression stiffness and thus should have at least theoretically contributed to 360° stiffness at the augmented level decreasing the bending-flexural forces that could result in loss of correction and material failure. The mean loss of correction in the Tomaoki series^16^ did not significantly differ from that in the series of anterior decompression and stabilization,^9^,^32^ while short posterior fixation without vertebral body augmentation^23^ for L_2_-L_4_ showed 5° loss of correction. No loss of correction was observed in this series.

The use of short instrumentation in combination with calcium phosphate grafting also saves mobile lumbar segments from fusion. The latter is of major importance for relatively young and active patients who have sustained lumbar fractures. The hypothesis of this study that balloon kyphoplasty with Calcibon grafting could directly reduce lost vertebral body height and restore posttraumatic kyphotic angle was justified from the short-term results of this study. The authors believe that the reduction with balloon at the site of the deformity is more etiological than the indirect reduction by means of distraction provided by instrumentation. Based on the preliminary results of this study the authors advocate the use of short instrumentation for thoracolumbar and lumbar burst fractures in combination with kyphoplasty with calcium phosphate and posterolateral fusion.
